# Hand-Assisted Laparoscopic Rectal Resection—Experience of a Tertiary Oncology Center

**DOI:** 10.3390/jcm14124097

**Published:** 2025-06-10

**Authors:** Beatriz Gonçalves, Beatriz Costeira, Filipa Fonseca, Francisco Cabral, André Caiado, Daniela Cavadas, João Maciel, Manuel Limbert

**Affiliations:** Department of General Surgery, Instituto Português de Oncologia de Lisboa Francisco Gentil (IPOLFG), R. Prof. Lima Basto, 1099-023 Lisboa, Portugal; bcosteira@ipolisboa.min-saude.pt (B.C.); ffonseca@ipolisboa.min-saude.pt (F.F.); fcabral@ipolisboa.min-saude.pt (F.C.); acaiado@ipolisboa.min-saude.pt (A.C.); ccavadas@ipolisboa.min-saude.pt (D.C.); jmaciel@ipolisboa.min-saude.pt (J.M.)

**Keywords:** hand-assisted laparoscopic surgery (HALS), rectal anterior resection (RAR), rectal cancer, rectal surgery, total mesorectal excision

## Abstract

**Background**: Hand-assisted laparoscopic surgery (HALS) is a possible approach for rectal anterior resection (RAR). However, evidence supporting this technique remains limited. This study aims to evaluate the perioperative and oncological outcomes of HALS for RAR at a single tertiary oncology center. **Methods**: A retrospective observational study was conducted using a prospectively maintained database. Patients with primary adenocarcinoma of the rectosigmoid junction and rectum who underwent HALS for RAR between 1 January 2013 and 31 December 2022 were included. All surgeries were performed by a dedicated colorectal team composed of three surgeons. **Results**: Among the 1911 surgeries for primary colorectal cancer performed, 469 met the inclusion criteria. The median age was 66 (57–74) years and 63% of the patients were male. Most tumors were cT3-4 (78.9%) and cN+ (71.2%), and neoadjuvant therapy was administered in 70.0% of cases. Low RAR was performed in 73.1% of cases, and an anastomosis was constructed in 95% of cases. The median operative time was 152 (135–180) min, and the conversion rate was 3.8%. Major morbidity occurred in 10.0% of cases, with 30-day and 90-day mortality rates of 0.9% and 1.3%, respectively. The overall anastomotic leak rate was 12.1%, with 9.0% early leaks and 3.1% late leaks. A complete/near-complete mesorectal excision was achieved in 89.6% of cases and an R0 resection in 96.2% of cases. With a median follow-up of 87 months, the locoregional recurrence rate was 2.5%, whereas the distant recurrence rate was 5.9%. The 5-year overall survival was 82.6%. **Conclusions**: When performed by experienced teams, HALS for RAR is safe and feasible and is associated with a short operative time, low conversion rate, minimal morbidity, and optimal oncologic performance.

## 1. Introduction

Over the past few years, rectal cancer (RC) treatment has undergone significant changes, notably with the advent of the watch-and-wait (WW) strategy [1] and the introduction of total neoadjuvant therapy (TNT) [2]. Despite these advances, surgery remains the cornerstone of treatment. Rectal anterior resection (RAR) with a total mesorectal excision (TME) [3] or partial mesorectal excision (PME) is the procedure of choice, whenever sphincter preservation is feasible. This is a technically demanding procedure that should be performed in high-volume centers to achieve optimal oncological outcomes with minimal morbidity [4].

Surgical techniques have also evolved. In recent decades, minimally invasive surgery (MIS) approaches have been developed and widely adopted in RC surgery. These approaches offer significant short-term advantages over open surgery and when performed at experienced centers, they ensure equivalent oncological outcomes [5,6,7,8,9]. Among these, laparoscopic abdominal surgery is the most commonly used, with increasing interest in transanal total mesorectal excision (Ta-TME) and robotic-assisted surgery. Hand-assisted laparoscopic surgery (HALS), though less frequently used, has been part of surgical practice since its introduction in the late 1990s [10,11,12,13]. Originally developed as a bridge to fully laparoscopic procedures, HALS involves the insertion of the surgeon’s hand into the abdominal cavity through a hand-port device, which maintains the pneumoperitoneum. This technique preserves tactile feedback and enhances laparoscopic instrument functionality, facilitating exposure, dissection, and hemostasis [14].

Evidence supporting the use of HALS in colorectal surgery is limited, as many studies have focused on small patient cohorts or combined results for colon and rectal surgeries, as well as oncological and benign conditions. Despite these limitations, HALS has demonstrated several advantages over open surgery like any MIS approach, such as a faster recovery of gastrointestinal function, reduced analgesic requirements [15], a shorter hospital stay [16], and lower hospital costs [17]. Additionally, an analysis of the American College of Surgeons National Surgical Quality Improvement Program (NSQIP) database revealed that patients undergoing HALS experienced lower overall complication rates, as well as fewer reoperations and readmissions when compared to those undergoing open surgery [18].

HALS has also been compared to pure laparoscopic surgery. The shorter learning curve is a key advantage. A Cochrane review [19] analyzing three randomized controlled trials (RCTs) [20,21,22] identified a lower conversion rate and a trend towards a shorter operative time for HALS. No significant differences were seen in terms of postoperative complications, the length of hospital stay, the number of retrieved lymph nodes, or mortality. A more recent meta-analysis that included 47 studies (five RCTs) [23] corroborated these findings, but found a longer incision required with HALS, which may increase the risk of incisional hernia. HALS has also shown particular benefits in obese patients [23] and in those with a narrow pelvis, a common challenge in male patients.

Despite these findings, RC remains underrepresented in studies addressing HALS, even in large single-center descriptive series. The Mayo Clinic reported a series comprising 1103 HALS procedures, of which only one-third were cancer-related and with rectal resections accounting for just 43% [24]. Similarly, Samalavicius et al. analyzed 467 colorectal cancer patients who underwent HALS, with only 181 cases involving an RAR [25].

At our institution, a tertiary oncology referral center, HALS was first introduced in 2007 for the management of left-sided colon and rectal cancer. For more than ten years, HALS with loop transverse colostomy has become the standard approach for rectal anterior resections.

This study aims to evaluate the perioperative and oncologic outcomes of HALS for RAR, including both short- and long-term results, thereby contributing to the growing evidence supporting minimally invasive techniques in rectal cancer surgery.

## 2. Materials and Methods

### 2.1. Ethics

The Ethics Committee of Instituto Português de Oncologia de Lisboa Francisco Gentil (IPOLFG) (UIC/1734, 7 March 2025) approved this study and granted it an exemption from having to obtain informed consent due to its retrospective design and non-interventional nature. Patients’ personal data were handled confidentially and in accordance with the General Data Protection Regulation (EU) [26]. An anonymized dataset supporting the findings of this study is available upon request from the authors.

Informed consent was obtained for the use of photographs related to the surgical procedure.

### 2.2. Study Design

We conducted a retrospective observational study using prospectively collected data from a single tertiary oncology institution. All consecutive patients operated on by the colorectal surgery unit between 1 January 2013 and 31 December 2022 were assessed for eligibility.

Patients meeting the following criteria were included in the analysis:

- 18-years or older;
- Biopsy-proven diagnosis of a primary adenocarcinoma of the rectosigmoid junction (RSJ) or the rectum;
- Submitted to HALS RAR.

The study is reported according to the STROBE guidelines [27].

### 2.3. Cancer Staging, Treatment, and Follow-Up Protocol

Patients routinely underwent total colonoscopy with tumor biopsy, chest computed tomography (CT) scan, abdominal CT or magnetic resonance imaging (MRI), and pelvic MRI. Endorectal ultrasound was reserved for cT1-T2N0 rectal cancer. Rectal tumors were categorized based on the MRI-measured distance from the anal verge to the inferior limit of the tumor as low (0–6 cm), medium (>6–11 cm), or high (>11–15 cm). RSJ tumors were defined by a distance >15 cm from the anal verge, for which resection implies a PME.

Patients with RSJ or RC were evaluated in a dedicated colorectal multidisciplinary meeting and were proposed for direct surgery or neoadjuvant therapy in the form of chemoradiotherapy (CRT), short-course radiotherapy (SCRT) or, since 2022, total neoadjuvant therapy (TNT), as outlined in Table 1. Until December 2021, all patients with node-positive disease (clinical or pathological) were considered for adjuvant chemotherapy. Since January 2022, chemotherapy has been preferentially administered preoperatively in combination with radiotherapy, as part of the TNT protocol, using a consolidation regimen. Patients with low-grade cT1 tumors of the mid or lower rectum and without adverse prognostic factors were offered transanal curative excision. Patients aged ≥ 80 years and/or with significant comorbidities requiring neoadjuvant therapy were offered SCRT followed by surgery. For patients with low rectal cancer selected for CRT or TNT with curative intent, response assessment was performed using clinical examination, MRI, and flexible sigmoidoscopy. The “watch and wait” (WW) protocol [1] was offered to patients with ycT0N0. The current treatment protocol for rectal cancer is outlined in Table 1.

The five-year surveillance following operative treatment includes regular CEA testing (every 3 months in the first 3 years, then every 6 months), clinical examination (every 6 months in the first 3 years, then annually), and annual thoraco-abdomino-pelvic CT. The colonoscopy is repeated at the end of the first year (or earlier if the preoperative colonoscopy was incomplete) and then, after 3 years.

### 2.4. Surgery Technique

HALS is the preferred technique for RAR at our center. An open approach is selected for patients with a history of major open abdominal surgery or locally advanced lesions requiring multivisceral resections (e.g., pelvic exenteration, sacrectomy, and peritonectomy). High RAR with partial mesorectal excision (PME) is the standard for RSJ and high rectal cancers, whereas low RAR with total mesorectal excision (TME) is the standard for mid and low rectal cancers. A diverting stoma, preferably a transversostomy, for anastomosis protection is offered to all patients with prior radiotherapy and those undergoing low RAR. A permanent stoma is preferred for frail patients with multiple comorbidities and for those with preoperative fecal incontinence. All procedures, either HALS or open surgery, were performed by a dedicated team of three colorectal surgeons.

#### Surgical Protocol

0. Day before surgery: Bowel preparation (metronidazole 750 mg PO 2id + neomycin 1000mg PO 3id + polyethylene glycol 3L PO + docusate enema 2id) for patients for whom an anastomosis and protective stoma are planned; stoma site marking;

1. Preoperative single shot of cefoxitin 2 gr EV 30 min before skin incision (repeated every 2 h of surgery);

2. Lithotomy position;

3. GelPort^®^ (Applied Medical, Rancho Santa Margarita, CA, USA) on the right flank, at the level of the umbilicus through a 5/6 cm horizontal incision (adapting the height to stoma site marking). Only the surgeon’s left hand is used through the GelPort^®^;

4. Trocar placement: 12 mm umbilical (assistant/camera), left (assistant), and right (surgeon’s right hand) iliac fossae. Pneumoperitoneum at 12 mmHg;

5. Abdominal exploration for liver metastases, ascites, or carcinomatosis;

6. Lymphadenectomy from the root of inferior mesenteric artery that is ligated after the emergence of the left colic artery (low tie). Ligation of inferior mesenteric vein at the same level (or at its origin if the colonic graft did not reach the pelvic cavity for a no-tension anastomosis);

7. Mobilization of left colon and splenic flexure;

8. Partial or total mesorectum excision (depending on the tumor location);

9. Rectal transection with a linear stapler. For ultralow tumors, transection is made below the pectineal line by a transanal approach, either manually or according to the method described by Limbert et al. [28];

10. Colon transection with purse string forceps after exteriorization through the GelPort^®^ site;

11. Specimen extraction. Intraoperative frozen section examination if there is uncertainty regarding distal or circumferential margins, which may warrant additional resection;

12. End-to-end or side-to-end intracorporeal mechanical anastomosis with circular stapler (double-staple technique). For ultralow tumors, a coloanal anastomosis was performed either manually or according to the method described by Limbert et al. [28];

13. Hemostasis. Pelvic passive drainage exiting through the left iliac fossa trocar;

14. Diverting proximal transversostomy (if indicated and after colonic mobilization) on the right flank through the GelPort^®^ site with partial closure of aponeurosis (leaving a ± 3 cm defect) and skin with intradermic suture;

15. Trocar site’s aponeurotic and skin defect closure.

The surgery is entirely performed with the surgeon having one hand inside the abdominal cavity through the hand device (left) and the other one (right) manipulating laparoscopic instruments.

**Figure 1:**
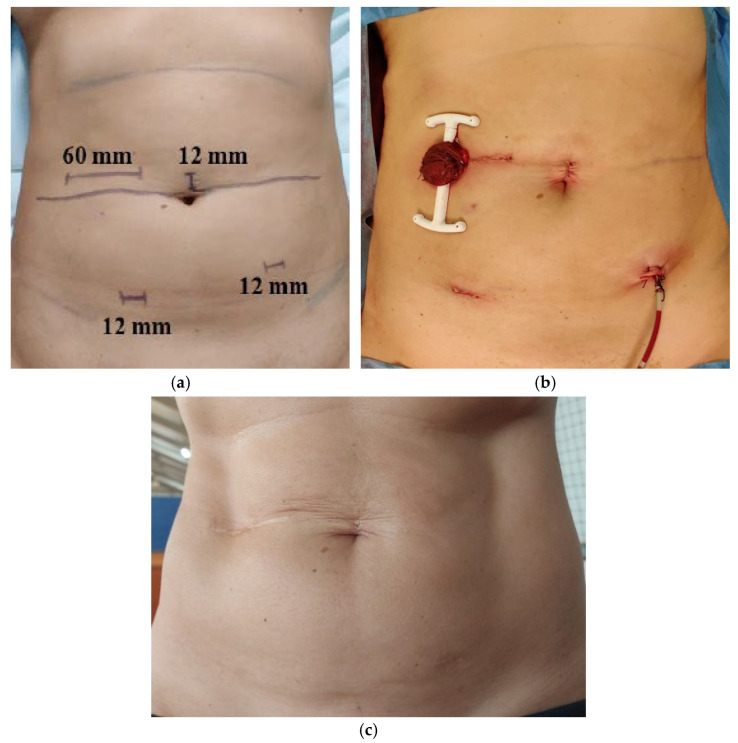
Preoperative skin marking for surgical incisions (**a**), immediate postoperative appearance (**b**), and five years after stoma closure (**c**).

Preoperative skin marking for surgical incisions and postoperative appearance (immediately after RAR and five years after stoma closure) are shown in Figure 1.

### 2.5. Variable and Outcome Definitions

All demographic and clinical data previously collected were validated by at least two members of the research team who worked together to review each patient’s record. Clinical data included pre-, intra-, and postoperative variables, as well as pathological details.

Patients’ comorbidities were evaluated using the Charlson Comorbidity Index (CCI) [29]. A CCI ≥5, associated with a 10-year mortality risk of 79% [29], was identified in a prospective cohort of 2531 patients with colorectal cancer as an independent risk factor associated with 5-year mortality [30] and was therefore considered a relevant cut-off for the analyses. Anemia was defined as hemoglobin <13 g/dL for male patients and <12 g/dL for female patients. Cancer staging was based on the TNM classification for colorectal carcinoma (AJCC 8th edition) [31]. Synchronous tumors were defined as those present within 6 months of the primary index tumor [32].

The Clavien–Dindo classification [33] was used to classify postoperative morbidity up to 30 days after surgery or during the index hospital admission (if longer than 30 days). Major morbidity was defined as Clavien–Dindo grade III or higher. Postoperative mortality was assessed at both 30 and 90 days.

Anastomotic leakage was defined according to the criteria established by the International Study Group of Rectal Cancer (ISREC) [34] and classified in two ways: by presentation time (early or late (≤ or >30 days after surgery, respectively)) and by severity (A, B, or C) [34]. Additionally, we further subdivided grade C into C1, for cases in which surgical intervention was required with anastomosis preservation, and C2, for cases in which surgical intervention was required without anastomosis preservation. Successfully restored bowel continuity was defined by the presence of gastrointestinal transit through a constructed, leak-free anastomosis.

Incisional hernias were identified based on both clinical assessment and imaging findings—follow-up CT scans were reviewed in cases for which no clinically diagnosed hernia was documented in the medical records. Incisional hernias were considered to be related to HALS if they occurred at the GelPort^®^ site (in patients without a stoma), at the previous stoma/GelPort^®^ site (in patients with a closed prior stoma), or at trocar sites. Laparotomy hernias (resulting from conversion to open surgery or following procedures performed using an open approach) and pure parastomal hernias (in patients with a permanent stoma) were excluded from this evaluation.

The Quirke classification [35] was used to assess the quality of the TME specimen. A positive circumferential or distal resection margin (CRM/DRM) was defined as <1 mm between tumor invasion and the mesorectal fascia or distal margin, respectively. Pathologic response after neoadjuvant treatment was based on Ryan’s grading system [36].

Recurrence data were collected from patients’ records. Local recurrence (LR) was defined by clinical, radiologic, and/or pathologic evidence of locoregional relapse (such as recurrence at anastomosis or previously treated tumor bed), whereas distant recurrence (DR) was defined by clinical, radiologic, and/or pathologic evidence of tumor spread to distant organs (such as the liver, lung, distant lymph nodes, or peritoneum).

Disease-free locoregional survival (DFLS) was defined as time from surgery to disease locoregional recurrence, and disease-free distant survival (DFDS) was defined as the time from surgery to disease distant recurrence. Overall survival (OS) was defined as time from surgery to death by any cause.

To estimate these long-term oncological outcomes, patients with the following characteristics were excluded:

- Previous tumors in the past 5 years (with the exception of non-melanoma skin cancer, well-differentiated thyroid tumors);
- Synchronous tumors (colorectal or other);
- Metastatic disease at diagnosis or at time of surgery;
- R2 resection;
- Metachronous tumors until 5 years after surgery.

### 2.6. Statistical Analysis

Patients were initially evaluated as a whole group. An exploratory analysis was carried out for all variables. Categorical data were presented as frequencies and percentages, and continuous variables as median and inter-quartile range (25th percentile; 75th percentile).

A comparison of the baseline characteristics between the HALS and the open-surgery groups was performed using the chi-squared test or Fisher’s exact test for categorical variables and using the non-parametric Mann–Whitney U test for continuous variables.

The Kaplan–Meier method was used to estimate OS, DFLS, and DFDS. For these long-term outcomes, duration of follow-up was estimated using the reverse Kaplan–Meier.

A two-tailed *p* value <0.05 was considered significant. Statistical analysis was performed using IBM SPSS Statistics (IBM Corporation, Armonk, New York, USA) version 25.0 and R version 4.2.0 [37].

## 3. Results

**Figure 2:**
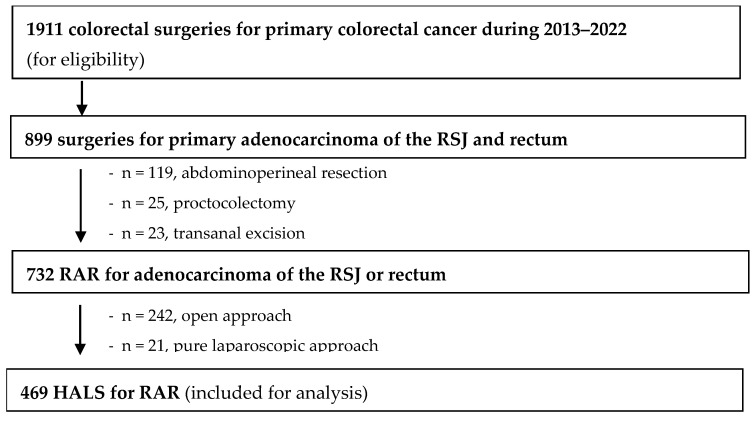
Study flowchart.

During the 10-year period from 1 January 2013 until 31 December 2022, a total of 1911 procedures for primary colorectal cancer were performed by our colorectal surgery unit. Of these, 469 were HALS for RAR by primary adenocarcinoma of the RSJ or the rectum and were therefore included for analysis. A patient selection flowchart is presented in Figure 2.

The median follow-up of the series was 70 (39–98) months.

### 3.1. Patients

Patient demographic and clinical characteristics are outlined in Table 2. The median age was 66 (57–74) years, and 63.1% were male. Most patients were ASA I-II (81.6%), and the median CCI was three (1–4), with 11.1% having a CCI score ≥5. A history of previous abdominal surgery was recorded in 17.1% of cases. Regarding tumor location, the majority were in the mid rectum (46.2%), followed by the upper rectum (23.7%), lower rectum (22.7%), and RSJ (7.5%). Most tumors were classified as cT3.4 (78.9%) and cN+ (71.2%). Metastatic disease at diagnosis was present in 20 patients (4.3%), with 11 cases involving the liver, six cases involving the lungs, one case involving distant lymph nodes, and two cases with metastases in more than one location.

Neoadjuvant therapy was administered in 70.0% of cases. Synchronous tumors were present in 1.9% of cases. A comparison between the baseline characteristics of the HALS group and the open-surgery one is described in Table S1 of Supplementary Materials. As expected, there was a greater proportion of patients submitted to open surgery who had previous abdominal surgery, cT3-T4 and cM1 tumors, synchronous tumors, and additional visceral resections.

### 3.2. Intraoperative Outcomes

Of the 469 HALS for RAR performed, 126 (26.9%) were high RARs with PME, while 343 (73.1%) were low RARs with TME. Anastomosis was performed in 95.1% of cases. Most of them were colorectal (87.4%), with a circular stapler (92.2%) being used in an end-to-end fashion (82.3%). A defunctioning stoma was created in 375 patients with anastomosis (84.0%), predominantly in cases of low RAR (99.1%). Most of these diverting stomas were transverse colostomies (97.6%). Additional visceral resections were performed in 2.8% of cases. The median operative time was 152 (135–180) min, and the median estimated blood loss was 75 (50–200) mL. Conversion to open surgery occurred in 3.8% of cases. The intraoperative outcomes are summarized in Table 3.

### 3.3. Postoperative Outcomes

The postoperative outcomes are described in Table 4. The major morbidity rate was 10.0%. The 30-day mortality rate was 0.9, while the 90-day mortality rate was 1.3%. All 90-day deaths corresponded to the in-hospital mortality (Clavien–Dindo grade V). The median length of hospital stay was 5 (4–7) days, and early hospital readmission occurred in 5.3% of cases.

Surgical site infection (SSI) was reported in 11.1% of patients, with 7.3% classified as superficial or deep SSI and 3.8% as organ/space SSI. Stoma-related complications occurred in 3.0% of cases, with just one case of stoma prolapse. The incidence of incisional hernia associated with HALS was 12.1%.

An anastomotic leak was observed in 54 patients (12.1%). Considering the timing of presentation, the early leak rate was 9.0%, while the late leak rate was 3.1%. Regarding severity, most patients were classified as type B (44.4%). Anastomotic leaks were more frequent in low RAR compared to high RAR surgeries (14.8% vs. 5.0%, *p* = 0.005) and in coloanal compared to colorectal anastomosis (32.1% vs. 9.2%, *p* < 0.005).

Most patients (40.7%) were managed conservatively with drainage and antibiotics, the majority of whom (20/22) had a defunctioning stoma in situ. Endoscopic treatment with EndoSponge^®^ was used in 13 patients (24.1%), all of whom diverted, while endoscopic clip placement was used in five patients (9.3%), two of whom diverted. Anastomotic takedown was required in 11 patients (20.4%). Leak resolution with anastomosis preservation was possible in 57.4% of cases and stoma closure in 48.1% of cases (with a median follow-up of 70 (39–98) months). Table 5 outlines anastomotic leak outcomes.

Therefore, bowel continuity was successfully restored in 89.0% of patients.

### 3.4. Short-Term Oncological Outcomes

Part of the short-term oncological outcomes pertain to the pathologic findings, which are summarized in Table 6. Pathological assessments of mesorectal quality excision were performed for 387 patients. Among these, a complete or near-complete mesorectal specimen was achieved in 89.6% of cases. The median number of lymph nodes excised was 15 (12–21). R0 resection was performed in 96.2% of cases. Positive DRMs were observed in only two patients (0.4), whereas positive CRMs were identified in 17 patients (3.6). A complete pathological response after neoadjuvant therapy was observed in 16.2% of cases.

Concerning adjuvant therapy, it was indicated for 319 patients (68.0%), and it was administered to 294 patients (62.7%), most frequently in the form of chemotherapy (60.3%). Adjuvant chemoradiotherapy was administered to 11 patients (2.4%). Due to surgical morbidity, the initiation of adjuvant treatment was delayed in 17 patients, and nine patients were unable to undergo any form of adjuvant treatment.

### 3.5. Long-Term Oncological Outcomes

A total of 421 patients were included to estimate long-term oncological outcomes. The median follow-up was 87 months (CI 95%; 83–91).

**Figure 3:**
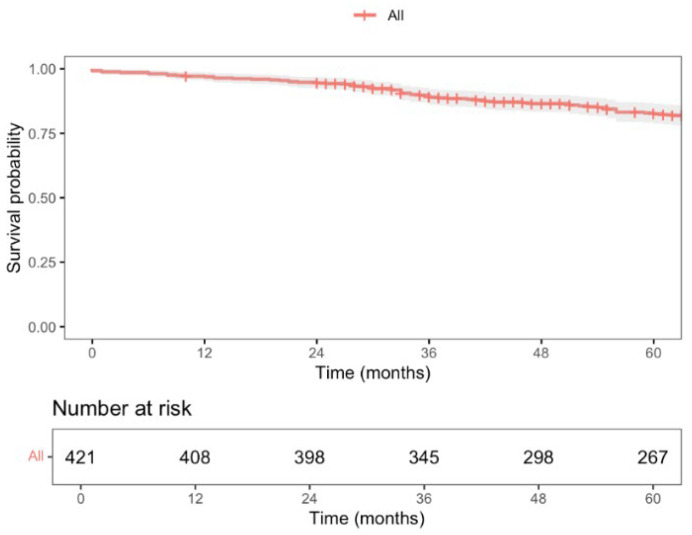
Overall 5-year survival of patients undergoing curative HALS for RAR for rectal cancer (stage I–III).

**Figure 4:**
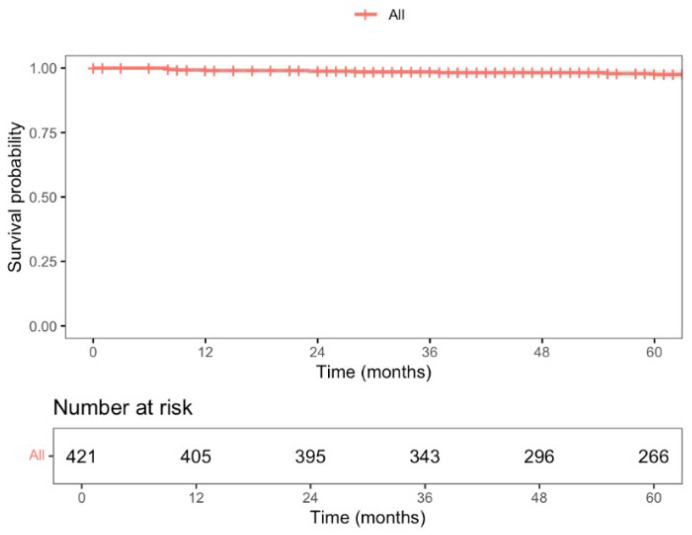
The 5-year disease-free locoregional recurrence (DFLR) rate of patients undergoing curative HALS for RAR for rectal cancer (stage I–III).

**Figure 5:**
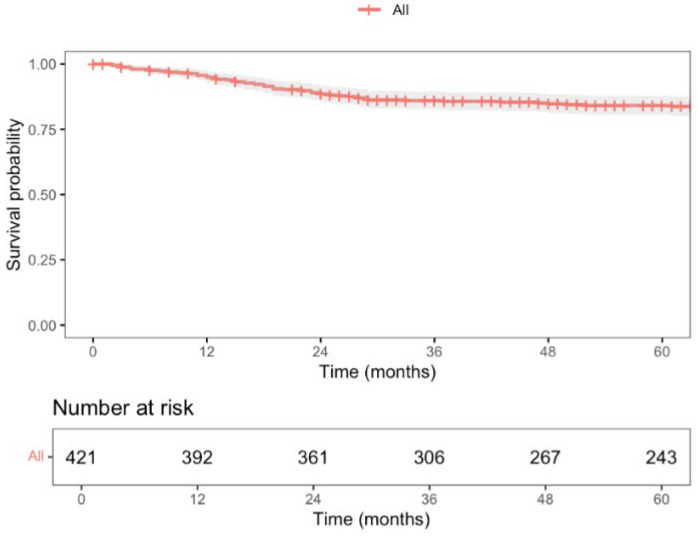
The 5-year disease-free distant recurrence (DFDR) rate of patients undergoing curative HALS for RAR for rectal cancer (stage I–III).

During this period, a total of 87 deaths were recorded, with a 5-year overall survival (OS) rate of 82.6% (95% CI; 78.8–86.5), as shown in Figure 3. Nine patients experienced locoregional recurrence (LR), and the 5-year LR rate was 2.5% (CI 95%; 0.8–4.1), as shown in Figure 4. Further, 66 patients experienced distant recurrence (DR), and the 5-year DR rate was 5.9% (CI 95%; 2.2–9.4), as shown in Figure 5.

## 4. Discussion

We present the experience of a tertiary oncology center with HALS for RAR, in the context of rectosigmoid junction and rectal cancer, focusing on intraoperative, postoperative, pathological, and oncological outcomes. Our findings support that HALS for RAR is associated with a short operative time, low conversion rate, low level of morbidity, and a low rate of locoregional recurrence.

The present study is, to the best of our knowledge, the largest series of HALS for RAR for cancer, with a case volume comparable to major trials evaluating the outcomes of laparoscopic, robotic, and Ta-TME surgeries. Table S2 of Supplementary Materials compares our perioperative results to key studies in the literature, including the COLOR II trial [5,6] (laparoscopic vs open), ALaCaRT trial [7] (laparoscopic vs open), ROLARR trial [8] (robotic vs laparoscopic), the study of Lee, Atallah et al. [38] (Ta-TME vs robotic), and the Ta-TME international registry (IR) [9].

Our series has one of the shortest operative times reported in the literature, with a median operative time of 152 min (135–180), which is shorter than that reported for laparoscopic [5,7,8] and robotic [8] groups, and even shorter than those reported for open groups in the COLOR II [5] and ALaCaRT [7] trials. While the operative time is not our primary focus, it reflects the standardization of surgical procedures and team expertise in high-volume centers. The conversion rate of 3.8% is notably low, with only Lee et al. [38] reporting lower rates (1.2% for transanal TME and 1.3% for robotic approaches).

Regarding postoperative outcomes, major morbidity was observed in 10.0% of patients, comparable to the 13.2% rate reported in the Ta-TME IR [9]. We report a 30-day mortality rate of 0.9%, which is within the range described in the literature for RAR (0.3–1.3%) [5,7,8,9,38]. The 90-day mortality rate, not reported in any of the studies, was 1.3%. The median hospital stay was 5 days, shorter than the 8–9 days reported in most trials [5,7,8,9]. Thirty-day readmission occurred in 5.3% of cases, comparable to retrospective studies of readmission after rectal surgery [39,40].

An anastomosis was created in 95.1% of cases, comparable to the 94.6% reported by Lee et al. [38]. The lower rates in the COLOR II [5] and ROLARR trial [8] likely reflect the inclusion of abdominoperineal resections (in 26% and 18% of cases, respectively). A diverting stoma was performed in 84% of cases, reflecting the high proportion of low RAR procedures and the use of neoadjuvant radiotherapy. Specifically in high RAR surgeries, the diverting stoma rate was 43.0%, which reflects not only patients who received neoadjuvant radiotherapy (23.8%), but also patients with comorbidities associated with higher risk of anastomotic complications, in which case an intraperitoneal leakage can have disastrous consequences. While most surgeons prefer ileostomies, we favor a protective colostomy using the proximal transverse colon. A meta-analysis by Gavriilidis et al. [41] reported a higher incidence of incisional hernias after colostomy reversal, but also found a greater rate of complications from high-output stomas in ileostomies. More recently, Yagyu et al. [42] showed that patients with a diverting ileostomy were more likely to need antidiarrheal drugs and outpatient IV fluids and experienced longer delays before stoma reversal compared to those with a diverting colostomy. Moreover, having a diverting ileostomy was identified as an independent risk factor for developing new-onset postoperative kidney disease. Our preference for colostomy is partly due to technical convenience—it can be easily created in the right flank at the GelPort^®^ site and reversed through the same incision—but more importantly, it reflects our aim to minimize the risks of dehydration, electrolyte imbalances, and renal complications associated with ileostomies.

The overall anastomotic leak rate was 12.1%, with 9.0% being early leaks and 3.1% late leaks. These rates are based on the ISREC definition [34] and include both clinical and subclinical events. This consensus, inclusive definition is only used in three of the five trials [5,9,38]. Moreover, we evaluate both early and late leaks, which are often underreported in reports in the literature, and only addressed Ta-TME IR [9]. In the latter, the overall leak rate was 15.7%, with 7.8% being early leaks, which is again comparable to our series. Notably, the rate of successfully restored bowel continuity, a frequently overlooked but important outcome for RAR patients, is 89.0%.

The incisional hernia rate is a potential disadvantage of this approach. In our series, with a median follow-up of 70 months, the incisional hernia rate associated with HALS was 12.1%. Not only is this rate lower than that traditionally reported for colorectal laparotomy (up to 30%) [43], but it is also lower than that reported in a subanalysis of the COLOR II trial [44], in which a 17.0% incisional hernia rate was found for the laparoscopy group.

Regarding oncologic outcomes, our series achieved a complete/near-complete mesorectal excision in 89.6% of cases, a rate nearly 10% lower than that reported in other series. Although mesorectum quality excision is not described in 17.5% of cases, the hand traction applied to the mesorectum in HALS surgery may contribute to this finding. Most importantly, these results do not appear to compromise curative resection or long-term oncologic outcomes, as described below. R0 resection was obtained in 96.2% of cases, and a positive CRM and DRM were seen in only 3.6% of cases and 0.4% of cases, respectively. It should be noted that the intraoperative frozen-section examination performed in cases of uncertainty regarding margins may influence the low rates mentioned herein.

With a median follow-up time of 87 months (CI 95%; 83–91), the 5-year locoregional recurrence (LR) rate was 2.5%, lower than the 5.0% reported at 3 years in the COLOR II trial for both open-surgery and laparoscopic groups [6]. The COREAN trial [45], which recently published its long-term results, reported a 10-year LR rate of 8.9% and 3.4% for open-surgery and laparoscopic approaches, respectively. Ta-TME studies by Lee et al. [38] and Lacy et al. [46] reported LR rates of 4.0% and 2.3%, respectively, but with a median follow-up of only 15 months. Our 5-year distant recurrence (DR) rate was 5.9%, as expected since DR is more influenced by tumor stage and systemic therapy response than by surgical local control. The 5-year overall survival (OS) was 82.6%, comparable to what is described in the literature. The COLOR II trial [6] reported a 3-year OS rate of 86.7% and 83.6% for laparoscopic and open-surgery groups, respectively, while the COREAN trial [45] showed a 10-year OS rate of 76.8% and 74.1% for each group.

The present study has several limitations. First and most importantly, its retrospective design carries an inherent risk of missing data and misclassification bias. In particular, the detection of incisional hernias may have been underestimated as imaging was routinely performed for oncologic surveillance rather than hernia detection, which may have led to an underreporting of asymptomatic cases. Second, abdominoperineal resections (APRs) were not included, as HALS was not the preferred approach for this technique. Given that APR is an integral part of rectal cancer management, future studies should aim to incorporate these patients. Lastly, the quality of mesorectal specimen was not documented in the pathology reports of 82 patients (17.5%), which is a considerable proportion for a tertiary oncology center.

Nevertheless, this high-volume series provides valuable insight into the perioperative as well as short- and long-term oncologic outcomes of HALS for RAR and contributes to the growing evidence supporting minimally invasive techniques in rectal cancer surgery.

## 5. Conclusions

HALS for RAR proved to be a safe and feasible MIS approach for the resectioning of the rectosigmoid junction and rectal cancer. It is associated with a short operative time, low conversion rate, minimal level of morbidity, high-quality oncological resection, and a notably low locoregional recurrence rate.

## Figures and Tables

**Table 1:** Current treatment protocol for RSJ and rectal cancer (since 1 January 2022).

	Direct Surgery	CRT + CT (TNT)	SCRT + CT (TNT)
High rectum (>11–15 cm) or RSJ	≤cT3a-b N0/N1 MRF− EMVI−	cT4b MRF+ (any cN, EMVI)	cT3c-d/T4a cN2 EMVI+ (MRF−)
Mid rectum (>6–11 cm)	≤cT3a-b N0 MRF− EMVI−	cT4b MRF+ (any cN, EMVI)	cT3c-d/T4a cN1/N2 EMVI+ (MRF−)
Low rectum * (0–6 cm)	cT1 N0 MRF− EMVI−	≥cT2 cN+ MRF+ EMVI+	

* For low rectum submitted to TNT—Reevaluation in 4–6 weeks; WW if ycT0N0. CRT: Chemoradiotherapy; CT: Chemotherapy; EMVI: Extramural vascular invasion; MRF: Mesorectal fascia; RSJ: Rectosigmoid junction; SCRT: Short-course radiotherapy; TNT: Totally neoadjuvant therapy; WW: Watch-and-wait.

**Table 2:** Patient demographic and clinical characteristics.

Patient and Tumor Characteristics		n = 469
Age at time of surgery in years, median (IQR)		66 (57–74)
Male sex, n (%)		296 (63.1)
ASA score, n (%)		
I–II		383 (81.6)
III-–V		86 (18.4)
CCI score		
Median (IQR)		3 (1–4)
≥5, n (%)		52 (11.1)
BMI		
Median (IQR), kg/m^2^		25.7 (23.3–28.1)
≥30 kg/m^2^, n (%)		63 (13.4)
Preoperative anemia, n (%)		125 (26.7)
Previous abdominal surgery, n (%)		80 (17.1)
Tumor location, n (%)		
Rectosigmoid junction		35 (7.5)
High rectum		111 (23.7)
Mid rectum		217 (46.2)
Low rectum		106 (22.6)
Staging, n (%)		
cT	T1–T2	99 (21.1)
	T3–T4	370 (78.9)
cN	N0	135 (28.8)
	N+	334 (71.2)
cM	M0	449 (95.7)
	M1	20 (4.3)
cTNM	I	70 (14.9)
	II	66 (14.1)
	III	313 (66.7)
	IV	21 (4.3)
Neoadjuvant therapy, n (%)		314 (70.0)
Isolated CRT		252 (53.7)
Isolated SCRT		51 (10.9)
TNT		6 (1.3)
Conversion CT + CRT/SCRT (cM1)		5 (1.1)
Synchronous tumors, n (%)		9 (1.9)
Prostate		2
Ependymoma		1
Gastric adenocarcinoma		1
Hepatocarcinoma		1
Thymoma		1
Pancreatic NET		1
Pulmonary NET		1
Primary-occult NET		1

BMI: Body mass index; CCI: Charlson comorbidity index; CRT: Chemoradiotherapy; CT: Chemotherapy; IQR: Interquartile range; NET: Neuroendocrine tumor; TNT: Totally neoadjuvant therapy.

**Table 3:** Intraoperative outcomes.

Intraoperative Outcomes		n = 469
RAR type, n (%)		
High RAR		126 (26.9)
Low RAR		343 (73.1)
Anastomosis, n (%)		446 (95.1)
Type, n (%)—n = 446	Colorectal	390 (87.4)
	Coloanal	56 (12.6)
Technique, n (%)—n = 446	Mechanic (circular stapler)	411 (92.2)
	Manual	31 (7.0)
	Missing	4 (0.8)
Configuration, n (%)—n = 446	End-to-end	367 (82.3)
	Lateral-to-end	78 (17.5)
	Missing	1 (0.2)
Protective stoma, n (%)—n = 446		375 (84.0)
Type, n (%)—n = 373	Transversostomy	366 (97.6)
	Ileostomy	9 (2.4)
By RAR type, n (%)	High RAR—n = 121	52 (43.0)
	Low RAR—n = 325	322 (99.1)
Terminal stoma, n (%)		23 (4.9)
Other visceral resections, n (%)		13 (2.8)
Appendicectomy		1
Enterectomy		2
Hepatic excisional biopsy		2
Mesenteric implant excision		1
Partial cystectomy		1
Partial prostatectomy		1
Splenectomy		1
Uni- or bilateral seminal vesical excision		2
Uni- or bilateral oophorectomy		2
Operative time in minutes, median (IQR)		152 (135–180)
Estimated blood loss in mL, median (IQR)		75 (50–200)
Conversion to open surgery, n (%)		18 (3.8)
Adhesions (previous surgery)		3
Bleeding		3
Urinary tract injury (or suspected)		3
Tumor invagination or perforation		2
Suspected carcinomatosis		1
Missing		5

IQR: Interquartile range; RAR: Rectal anterior resection.

**Table 4:** Postoperative outcomes.

Postoperative Outcomes	n = 469
Major morbility (Clavien-Dindo ≥ III), n (%)	47 (10.0)
IIIa	12 (2.6)
IIIb	26 (5.5)
IVa	2 (0.4)
IVb	1 (0.2)
V	6 (1.3)
Reoperation (≤30 days), n (%)	30 (6.4)
Postoperative mortality, n (%)	
30 days	4 (0.9)
90 days	6 (1.3)
Length of hospital stay in days, median (IQR)	5 (4–7)
Hospital early readmission (≤30 days), n (%)	25 (5.3)
Overall anastomotic leak, n (%)—n = 446	54 (12.1)
SSI, n (%)	52 (11.1)
Superficial and deep incisional	34 (7.3)
Organ or space	18 (3.8)
Stoma-related complications, n (%)—n = 396	12 (3.0)
Mucocutaneous dehiscence	6
Perforation	3
Stenosis	2
Prolapse	1
Incisional hernia related to HALS, n (%)	57 (12.1)
Previous stoma/GelPort^®^ site	53
GelPort^®^ site without stoma	1
Trocar site	3

HALS: Hand-assisted laparoscopic surgery; IQR: Interquartile range; SSI: Surgical site infection.

**Table 5:** Anastomotic leak details.

Anastomotic Leak	n = 446	*p*-Value
Overall, n (%)	54 (12.1)	
Timing of presentation, n (%)		
Early (≤30 days)	40 (9.0)	
Late (>30 days)	14 (3.1)	
Severity grading, n (%)—n = 54		
A—without active therapeutic intervention	5 (9.3)	
B—nonsurgical intervention	24 (44.4)	
C—C1—surgical intervention, with anastomosis preservation	14 (25.9)	
—C2—surgical intervention, without anastomosis preservation	11 (20.4)	
By surgery type		
High RAR—n = 121	6 (5.0)	
Low RAR—n = 325	48 (14.8)	0.005
By anastomosis type		
Colorectal—n = 390	36 (9.2)	
Coloanal—n = 56	18 (32.1)	<0.005
Management, n (%)—n = 54		
No treatment	5 (9.3)	
Conservative (drainage and antibiotic)	22 (40.7)	
Protective stoma	20	
Endoscopic negative pressure therapy (EndoSponge^®^)	13 (24.1)	
Protective stoma	13	
Endoscopic clip	5 (9.3)	
Protective stoma	2	
Anastomotic takedown	11 (20.4)	
After failure of conservative or endoscopic treatment	2	
Leak resolution with anastomosis preservation, n (%)—n = 54	31 (57.4)	
Stoma closure (in those with leak), n (%)—n = 54	26 (48.1)	

RAR: Rectal anterior resection.

**Table 6:** Pathologic outcomes.

Pathologic Outcomes	n = 469
Mesorectum quality, n (%)—n = 387	
Complete	285 (73.6)
Near-complete	62 (16.0)
Incomplete	40 (10.4)
(not described)	(82)
Lymph node count, median (IQR)	15 (12–21)
Resection radicality, n (%)	
R0	451 (96.2)
R1	18 (3.8)
R2	0 (0.0)
Positive resection margins, n (%)	
CRM	17 (3.6)
DRM	2 (0.4)
Tumor regression grade, n (%)—n = 314	
0 (complete response)	51 (16.2)
1 (near-complete response)	67 (21.3)
2 (partial response)	136 (43.3)
3 (poor/no response)	53 (16.9)
Not described	7 (2.3)
(y)pTNM, n (%)	
0	56 (11.9)
I	155 (33.1)
II	112 (23.9)
III	121 (25.8)
IV	25 (5.3)

CRM: Circumferential resection margin; DRM: Distal resection margin; IQR: Interquartile range.

## Data Availability

The data presented in this study are available on request from the corresponding authors due to ethical reasons.

## Supplementary Materials

The following supporting information can be downloaded at: https://www.mdpi.com/article/10.3390/jcm14124097/s1, Table S1: Baseline characteristics in the HALS and open RAR groups; Table S2: Perioperative results of rectum anterior resection—literature comparison.
