# Supplementary material for: Hand-Assisted Laparoscopic Rectal Resection—Experience of a Tertiary Oncology Center
Source: J Clin Med. 2025 Jun 10;14(12):4097. doi: 10.3390/jcm14124097 (PMC12194001; doi:10.3390/jcm14124097)
Supplement: Supplementary file 1 [file jcm-14-04097-s001.zip › jcm-3643150-supplementary.pdf]

Supplementary Materials

Table S1: Baseline characteristics in the HALS and open RAR groups.

Baseline characteristics	HALS RAR	Open RAR	*p*-value
Age at surgery in years, median (IQR)	66 (57-74)	67 (59-75)	0.139
Male sex, n (%)	296 (63.1)	141 (59.0)	0.287
ASA score III–IV, n (%)	86 (18.4)	73 (30.9)	<0.005
CCI score ≥ 5, n (%)	32 (14.0)	52 (11.1)	0.267
BMI ≥30 kg/m2, n (%)	63 (13.5)	44 (18.6)	0.077
Preoperative anemia, n (%)	125 (26.7)	110 (46.0)	<0.005
Previous abdominal surgery, n (%)	80 (17.1)	88 (36.8)	<0.005
Tumor location, n (%)			
Rectosigmoid junction	35 (7.5)	31 (13.0)	
High rectum	111 (23.7)	50 (20.9)	0.078
Mid rectum	217 (46.3)	99 (41.4)	
Low rectum	106 (22.6)	59 (24.7)	
cTNM staging, n (%)			
cT3–4	370 (78.9)	207 (87.7)	0.007
cN+	334 (71.2)	184 (77.6)	0.076
cM+	20 (4.3)	45 (18.8)	<0.005
Neoadjuvant therapy, n (%)	314 (67.0)	173 (72.4)	0.146
Synchronous tumors, n (%)	9 (1.9)	13 (5.4)	0.019
Additional visceral resections, n (%)	13 (2.8)	87 (36.4)	<0.005

ASA: American Society of Anesthesiologists; BMI: Body Mass Index; CCI: Charlson Comorbidity Index; IQR: Interquartile range; HALS: Hand-assisted laparoscopic surgery; RAR: Rectal anterior resection.

Table S2: Perioperative results of rectum anterior resection—literature comparison.

	**IPOLFG**	**COLOR II [5,6]**		**AlaCaRT [7]**		**ROLARR [8]**		**Lee, Atallah et al. [38]**		**Ta-TME IR [9]**
**Approach**	**HALS**	**Lap**	**Open**	**Lap**	**Open**	**Lap**	**Rob**	**Ta-TME**	**Rob**	**Ta-TME**
N	469	699	345	238	237	234	237	226	370	1594
Low RAR, n (%)	343 (73.1)	418 (60)	230 (67)	212 (89)	211 (90)	158 (67.5)	159 (67.1)	226 (100)	370 (100)	1411 (91.6)
≥cT3, %	78.9	-	-	63	66	-	-	78	78.9	69
Neoadjuvant CRT, %	68.9	59	58	50	49	46.2	46.8	70.7	69.2	56.1
Op time (minutes), median	152	240	188	210	190	261	298.5	189.9	189.1	252
Conversion rate, %	3.8	16	NA	9	NA	12.2	8.1	1.3	1.1	6.4
Anastomosis, %	95.1	85.2		-		77.5		94.6		Only patients w/ anastomosis
Clavien-Dindo ≥III, %	10.0	-		-		-		-		13.2
Mortality, %	0.9 (30d)<br>1.3 (90d)	1.3 (28d)		0.6 (30d)		0.9 (30d)		0.3 (30d)		0.6 (30d)
Overall AL, %	12.1	-	-	7 (clinical AL, timing?)		9.9 (6 months)	12.2	-	-	15.7
Early AL, %	9.0	13	10			-	-	11.1	9.5	7.8
Hospital stay (days), median	5	8	9	8	8	8.2	8	-	-	8
Lymph node count, median	15	13	14	-	-	24.1	23.2	16.1	16.8	16
Complete/ near complete mesorectum, %	89.6	98	98	97	99	91.7	89.4	99.1	99.2	96.6
CRM +, %	3.6	10	10	7	3	6.3	5.1	6.3	6.2	3.9
DRM +, %	0.4	-	-	1	1	-	-	1.8	0.3	0.6

AL: Anastomotic leak; CRM: Circumferential resection margin; CRT: Chemoradiotherapy; DRM: Distal resection margin; HALS: Hand-assisted laparoscopic surgery; RAR: Rectal anterior resection; Ta-TME: Transanal total mesorectal excision.
